# 

**Published:** 1861-05

**Authors:** 


AMERICAN.

A Treatise on Human Physiology. By John C. Dalton, Jr., M.D., etc. Second edition. 8vo., pp. 690. Philadelphia: Blanchard & Lea, 1861. (From the publishers.)

Electro-Physiology and Electro-Therapeutics, showing the Best Methods for the Medical Uses of Electricity. By Alfred C. Garratt, M.D. Second edition. 8vo., pp. 716. Boston: Ticknor & Fields, 1861. (From the author.)

A Warning to Fathers, Teachers, and Young Men, in relation to a Fruitful Cause of Insanity and other Serious Disorders of Youth. By W. S. Chipley, M.D., etc. 12mo., pp. 188. Lexington, 1861. (From the author.)

Proceedings of the Academy of Natural Sciences of Philadelphia. 8vo., pp. 63. 1861. (From the Secretary.)

Annual Report of the Registrar of Births, Marriages, and Deaths, of the State of Kentucky, for the Year ending December 31, 1859. 8vo., pp. 152. Frankfort, 1861. (From Dr. Bemiss.)

Report on Morbus Coxarius, or Hip Disease. By Lewis A. Sayre, M.D. Reprint. 8vo., pp. 94. Philadelphia, 1860. (From the author.)

Annual Report of the Board of Trustees and Officers of Longview Asylum, to the Governor of the State of Ohio, for the Year 1860. Pamphlet. pp. 34. Columbus, 1861. (From Dr. Langdon.)

A Summary of the Anatomy and Physiology of the Placenta. By John O'Reilly, M.D., etc. Reprint. pp. 45. New York, 1861. (From the author.)

FOREIGN.

A Book about Doctors. By J. Cordy Jeaffreson. 12mo., pp. 409. New York, 1861.

Traité Pratique de l'Art des Accouchements. Par le Dr. Chailly-Honoré. Fourth edition. 8vo. Paris: Baillière, 1861.

A Treatise on Fever; or, Selections from a Course of Lectures on Fevers. By Robert D. Lyons, M.D., etc. 8vo., pp. 362. Philadelphia: Blanchard & Lea, 1861. (From the publishers.)

Foundation for a New Theory and Practice of Medicine. By Thomas Inman, M.D. Second edition. 8vo., pp. 528. London: Churchill, 1861.

A Treatise on Diseases of the Joints. By Richard Barwell, F.R.C.S. 8vo., pp. 469. London: Churchill, 1861.

Transactions of the Epidemiological Society of London. Part I. vol. I. 8vo., pp. 128. London: Richards, 1861. (From the Society.)

Description of Cases Communicated to the Pathological Society of London, during the Session 1859–60. By John Ogle, M.D. 8vo., pp. 49. (From the author.)

Anatomy, Descriptive and Surgical. By Henry Gray, F.R.S. Royal 8vo., second edition. London: Parker & Son, 1861.

Du Diagnostic des Maladies des Yeux à l'aide de l'Ophthalmoscope, et de leur Traitement. Par le Dr. J. D. Guérineau. 8vo. Paris: P. Asselin, 1861.

The Eastern, or Turkish Bath: Its History, Revival in Britain, and Application to the Purposes of Health. By Erasmus Wilson, F.R.S. Fcap 8vo. London: Churchill, 1861.

The Medical Critic and Psychological Journal. Edited by Forbes Winslow, M.D. No. 1. London: Davies, 1861. (From the editor.)

A Practical Treatise on Diseases of the Eye. By William Mackenzie, M.D. Fourth edition, revised and much enlarged. 8vo. London, 1861.

